# Nutrient Intake in Italian Infants and Toddlers from North and South Italy: The Nutrintake 636 Study

**DOI:** 10.3390/nu6083169

**Published:** 2014-08-08

**Authors:** Gian Vincenzo Zuccotti, Cristina Cassatella, Ambra Morelli, Maria Cristina Cucugliato, Giuseppina Catinello, Valeria del Balzo, Lucia Guidarelli, Carlo Agostoni, Chiara Mameli, Ersilia Troiano, Giorgio Bedogni

**Affiliations:** 1 Department of Pediatrics, Luigi Sacco Hospital, University of Milano, Via G.B. Grassi 74, 20157 Milano, Italy; E-Mails: gianvincenzo.zuccotti@unimi.it (G.V.Z.); mameli.chiara@hsacco.it (C.M.); 2 Azienda Sanitaria Locale, Corso Italia 19, 20122 Milano, Italy; E-Mail: ccassatella@asl.milano.it; 3 Ospedale San Carlo, Via Ospedale 21, 20037 Paderno Dugnano, Italy; E-Mail: ambramorelli@alice.it; 4 Italian Association of Dietitians, Vicolo S. Silvestro 6, 37122 Verona, Italy; E-Mails: mcristina.c@alice.it (M.C.C.); giucati@libero.it (G.C.); e.troiano@icloud.com (E.T.); 5 Human Nutrition Unit, Department of Experimental Medicine, La Sapienza University, Viale Regina Elena 324, 00161 Roma, Italy; E-Mail: valeria.delbalzo@uniroma1.it; 6 Nutrition and Consumers Information Office, Directorate of Hygiene, Food Safety and Nutrition, Ministry of Health, Viale G. Ribotta 5, 00144 Roma, Italy; E-Mail: l.guidarelli-esterno@sanita.it; 7 Department of Clinical Sciences and Community Health (DISCCO) and Fondazione IRCCS Cà Granda-Ospedale Maggiore Policlinico, University of Milano, Via Commenda 9, 20122 Milano, Italy; E-Mail: carlo.agostoni@unimi.it; 8 Liver Research Center, AREA Science Park, Strada Statale 14 - km 163.5, 34012 Basovizza, Italy; 9 International Center for the Assessment of Nutritional Status (ICANS), University of Milano, Via Botticelli 21, 20133 Milano, Italy

**Keywords:** epidemiology, cross-sectional study, anthropometry, food intake, weighed food dietary record, infants, toddlers

## Abstract

We performed a cross-sectional study to compare the intake of energy, macronutrients, fiber, sodium and iron and the anthropometric status of infants and toddlers living in North (Milano) and South Italy (Catania). Nutrient intake was evaluated using a 7-day weighed food record. Out of 400 planned children aged 6 to 36 months, 390 (98%) were recruited, 189 in Milano and 201 in Catania. The mean (standard deviation) age was 17 (9) months in Milano and 17 (10) months in Catania. Anthropometry, energy intake and macronutrient intake were similar in Milano and Catania. However, iron intake was 27% lower and fiber intake 16% higher in Milano than in Catania. Despite normal anthropometry and energy intake, in the pooled sample there was a high intake of proteins, simple carbohydrates, saturated fats and sodium, and a low intake of iron and fiber compared to Italian reference values. This is the first study to report the macro- and micro-nutrient intake of children aged <12 months using the 7-day weighed food record and one of the very few studies that have employed such reference method in children from the general population.

## 1. Introduction

There is a general agreement that the promotion of healthy lifestyles during the first years of life is central to the prevention of obesity and its complications [1]. However, evidence linking infant nutrition with adult obesity is presently lacking [2], a situation that is partly attributable to the unavailability of good-quality data on the nutrient intake of infants and toddlers [3,4,5]. Besides requiring accurate instruments and experienced operators in all ages of life [6,7], a detailed assessment of nutrient intake is more challenging in preschoolers than in schoolchildren because the former cannot be enrolled and studied at school [3,4]. Under the Italian National Health System, children are cared for by family pediatricians [8]. This offers the possibility to control the logistic barrier associated with the enrollment of infants and toddlers but does not attenuate the need of using the best available instruments to assess food intake [5,9].

The 7-day weighed food-record (7DWFR) is the accepted reference method for the assessment of nutrient intake [6,7] and offers accurate estimates of macronutrient intake compared to direct chemical analysis [10,11]. A recent systematic review concluded that weighed records are needed to obtain more accurate estimates of nutrient intake in children, especially when micronutrient intake is of interest [5]. Iron and sodium are especially important micronutrients for infants and toddlers, the first because of the frequency of iron-deficiency anemia [12,13] and the second because of its potential role in later cardiovascular disease [13,14]. To our knowledge, no study so far has evaluated the macro- and micro-nutrient intake of children aged <12 months using the 7DWFR even if the macronutrient intake of children participating to a randomized controlled trial (RCT) has been recently evaluated using the 3-day weighed food-record (3DWFR) [15,16]. An increasing north to south gradient of obesity is known to exist within Europe [17] and a similar gradient has been reported among Italian children aged ≥2 years [18,19]. It is however unknown whether such a gradient is present in the first years of life.

The aim of the present study, known as Nutrintake 636 (NI636), was to compare the intake of energy, macronutrients, fiber, sodium and iron and the anthropometric status of infants and toddlers living in North (Milano) and South Italy (Catania).

## 2. Experimental Section

### 2.1. Study Design

NI636 is a cross-sectional study aimed at comparing: (1) the intake of energy (E) (main outcome); (2) the intake of total carbohydrates (CHO), simple carbohydrates (CHOS), total fats (FAT), saturated fats (SFA), proteins (PRO), fiber, sodium and iron (secondary outcomes) and; (3) the weight, length/height and body mass index (BMI) (secondary outcomes) of Italian infants and toddlers living in North (Milano, Lombardy) and South (Catania, Sicily) Italy. NI636 children were enrolled among the infants and toddlers cared for by a convenience sample of Italian family pediatricians. NI636 pediatricians were recruited by the principal investigator (GVZ) by word of mouth at scientific meetings on infant nutrition. Inclusion criteria were: (1) children aged 6.0 (±0.5), 9.0 (±0.5), 12.0 (±1.0), 18.0 (±1.0), 24.0 (±1.0) or 36.0 (±1.0) months and, (2) parents with a good command of the Italian language. Exclusion criteria were any acute illnesses (e.g., influenza) or chronic diseases (e.g., food allergy) known to interfere with normal nutrition and growth. The study protocol was approved by the Ethical Committee of the Luigi Sacco Hospital (Milano, Italy) and the parents of the children gave their written consent to participate.

### 2.2. Sample Size

We aimed at detecting a median difference of 100 kcal·day^−1^ of E between Milano and Catania with a power ≥80% and a type I error rate of 5% taking into account the effects of sex and age. 100 kcal·day^−1^ of E (10%) was considered the minimum between-place biologically relevant difference on the basis of an expected median intake of 1000 kcal·day^−1^ in the pooled sample. The same difference of 10% was considered the minimum between-place biologically relevant difference for the nutrients (CHO, CHOS, FAT, SFA, PRO, fiber, sodium and iron). Our experience in validating the 7DWFR against chemical analysis suggests that a threshold of 10% is a reasonable choice to guard against instrumental error [10,11]. To calculate sample size, we applied Monte Carlo simulation to a multivariable median regression model having E (kcal·day^−1^) as response variable and the following predictors: (1) a between-place difference of 100 kcal·day^−1^ of E, (2) a between-sex effect ≥20% of median E and, (3) a one-degree fractional polynomial (FP) of age modelling the continuous energy-age relationship [20,21,22]. From the multiple scenarios generated by Monte Carlo simulation, we obtained that we had to enroll 400 children, 200 in Milano and 200 in Catania. On the basis of previous experience with similar epidemiological studies [23,24], we estimated that we had to recruit 600 subjects to obtain a number of 400 subjects.

### 2.3. Recruitment Procedure

Each NI636 pediatrician sent an anonymized list of potentially eligible children to the methodologist (GB). Such list included a unique pediatrician code, a unique patient number and the sex and birthdate of each child. The children from Milano were pooled together and the same was done for the children from Catania. From these pools, 300 children were randomly selected per place. Within-place pooling was done in order to control for the within-place between-pediatrician variability. We tried to obtain a similar between-pediatrician distribution of age and sex but this was not possible for children aged 6 and 9 months, for whom some pediatricians contributed more subjects both in Milano and Catania. The anonymized list with the randomly selected children was returned to the pediatricians who decoded it and shared it with the dietitians (CC, AM, MCC and GC).

### 2.4. Family Data

The dietitians collected the following sociodemographic and anthropometric data for both the parents of NI636 children: school degree, profession, physical activity level and self-reported weight and height. The dietitians also collected the pre-pregnancy weight and the weight increase during pregnancy for the mothers of NI636 children. BMI was calculated and classified according to the World Health Organization (WHO) [25].

### 2.5. Anthropometry

Anthropometric measurements were performed by the dietitians following international guidelines [26]. Weight was measured using a medical-certified SECA 384/385 baby scale (SECA, Hamburg, Deutschland). Supine length was measured using a medical-certified SECA 417 measuring board in children aged <24 months (SECA, Hamburg, Deutschland). Height was measured using a medical-certified SECA 213 portable stadiometer in children aged ≥24 months (SECA, Hamburg, Deutschland). Standard deviation scores (SDS) of weight, length, height and BMI were calculated using WHO reference data for children [27].

### 2.6. Food Intake

Food intake was evaluated by the dietitians using a 7DWFR, *i.e.*, the accepted reference method for the assessment of food intake [6,7]. The 7DWFR was administered to the parents of the children during a first encounter lasting 30 to 45 min. When the 7DWFR was returned one week later, the dietitians discussed its contents with the parents, asked for clarifications when needed, and wrote their comments in an apposite section of the 7DWFR. The second encounter also lasted from 30 to 45 min. Detailed recipes were always obtained for foods eaten at home. Menus with detailed recipes were obtained from the canteen staff for children eating at the kindergarten. Single ingredients were always recorded on the 7DWFR. We did not evaluate breast milk intake for two reasons. The first reason is that a random sample of 10 mothers interviewed during a pilot study uniformly expressed concern about the need of regularly weighing their children before and after breastfeeding. The second reason is the lack of good-quality data to model breast milk composition of Italian mothers.

### 2.7. Data Entry

The dietitians recorded all data on anonymized case report forms and entered them into a specifically developed web-based application. Such application allowed the entry of new foods and was accessible using the HyperText Transfer Protocol over Secure Socket Layer (HTTPS).

### 2.8. Food Composition

Food records entered into the database were resolved into a working foodlist of unique items on the basis of food name and brand. Each item of the working foodlist was manually checked by the food database manager (ET) and by the methodologist and linked with food composition data to produce a definitive foodlist. The definitive foodlist was generated and linked with food composition data and 7DWFRs by specifically developed Stata 13.1 programs (Stata Corp., College Station, TX, USA). Food composition data was predominantly obtained from the Food Composition Database for Epidemiological Studies in Italy [28] and from a specifically developed infant food database. When a nutrient was not available in such databases, we obtained it: (1) from another Italian food composition database [29] or, (2) from the ingredients specified on the food label.

### 2.9. Statistical Analysis

Descriptive statistics are reported as 50th (P_50_), 25th (P_25_) and 75th (P_75_) percentiles for non-Gaussian distributed variables and as mean and standard deviation (SD) for Gaussian-distributed variables. Discrete variables are reported as counts and percentages. Between-place comparisons of age were performed using Student’s unpaired *t*-test and between-place comparisons of sex using Fisher’s exact test. Exact logistic regression was used to test whether the odds of breastfeeding (discrete: 0 = not breastfed; 1 = breastfed) differed between place (discrete: 0 = Catania; 1 = Milano) with and without correction for age (continuous: 6, 9, 12, 18, 24 and 36 months) [30]. The contribution of place to the outcomes of interest (E, CHO, CHOS, FAT, SFA, PRO, fiber, iron, sodium, weight, length/height and BMI) was evaluated using multivariable quantile regression models employing P_25_, P_50_ and P_75_ as response variable and place (discrete: 0 = Catania; 1 = Milano), sex (discrete: 0 = female; 1 = male) and age (continuous: 6, 9, 12, 18, 24 and 36 months) as predictors [22]. One-degree fractional polynomials (FP) were used to account for non-linear relationships of the outcomes with age [21]. Because most of the between-place differences were negligible on biological grounds (≤10%, see *Sample Size*), we pooled together the children in Milano and Catania and refitted the regression models using only sex and age as predictors. Such models were then used to estimate nutrient intake in the pooled sample. Statistical analysis was performed using Stata 13.1 (Stata Corp., College Station, TX, US).

## 3. Results

### 3.1. Characteristics of NI636 Children

NI636 was performed between 15 September 2012 and 24 February 2013 and recruited 390 (98%) out of 400 planned children, 189 in Milano and 201 in Catania. NI636 children were enrolled by 11 out of 20 family pediatricians (4 in Milano and 7 in Catania) contacted by the principal investigator (see *Recruitment Procedure*). All NI636 children were Caucasians and their distribution by age is given in Table 1.

**Table 1:** Within-place distribution of age and family characteristics of NI636 children.

Variable	Milano (%)	Catania (%)
Age class		
6 months	20	13
9 months	13	18
12 months	16	22
18 months	16	18
24 months	17	16
36 months	18	13
School degree—fathers ^a^		
Elementary	0	3
Middle	13	39
High	43	43
University	44	15
School degree—mothers ^b^		
Elementary	0	1
Middle	7	35
High	38	39
University	55	25
Profession—fathers ^b^		
Unemployed	1	7
Employed	64	60
Freelance	35	32
Retired	0	1
Profession—mothers		
Unemployed	10	61
Employed	74	30
FreelanceRetired	160	90
BMI class (WHO)—fathers		
Underweight	0	1
Normal	57	38
Overweight	35	44
Obesity class 1	7	15
Obesity class 2	1	1
Obesity class 3	0	1
BMI class (WHO)—mothers		
Underweight	9	6
Normal weight	75	65
Overweight	13	21
Obesity class 1	2	7
Obesity class 2	0	0
Obesity class 3	1	1
Physical activity—fathers		
None	53	72
Light	16	12
Moderate	28	12
Heavy	3	4
Physical activity—mothers ^c^		
None	73	87
Light	13	9
Moderate	13	4
Heavy	1	0
Cigarette smoking—fathers		
No	69	57
Yes	31	43
Cigarette smoking—mothers		
No	82	77
Yes	18	23

^a–c^: Not available for 2, 1 and 3 parents. The entries in each column sum to 100%. WHO = World Health Organization.

The within-age between-pediatrician distribution of NI636 children is given in Table A1 of the Appendix. The mean (SD) age was 17 (9) months in Milano and 17 (10) months in Catania (*p* = 0.3, unpaired Student’s *t*-test). Males made up 50% of the sample in Milano and 49% in Catania (*p* = 1.0, Fisher’s exact test).

A total of 81 children (21%) were being breastfed, 42 in Milano and 39 in Catania. The frequency of breastfeeding was 43%, 30%, 16%, 9%, 1% and 1% at 6, 9, 12, 18, 24 and 36 months, respectively. The odds of being breastfed were similar in Milano and Catania with (odds ratio (OR) = 1.2, exact 95% confidence interval (CI) 0.7 to 2.0, exact *p* = 0.6) and without correction for age (OR = 1.2, exact 95% CI 0.7 to 2.2, exact *p* = 0.5). No child was exclusively breastfed, *i.e.*, breastfed without any additional food, drink or water [31].

Ten percent of children in Catania and 51% in Milano were eating their lunch at the kindergarten from Monday to Friday. One percent of children in Catania and 27% in Milano were practicing structured exercise, mostly in water (100% in Catania and 80% in Milano). All children in Milano and 92% in Catania practiced such structured exercise once a week.

### 3.2. Characteristics of the Parents of NI636 Children

The characteristics of the parents of NI636 children are given in Table 1. In line with the available epidemiological studies in Italy, parents in Milano had higher school degrees than those in Catania. The number of freelance fathers was similar in Milano and Catania (35% *vs.* 32%) but there were more freelance mothers in Milano than in Catania (16% *vs.* 9%). A similar number of freelance mothers were caring for 6-month infants in Milano and Catania (19% *vs.* 16%). The higher frequency of overweight and obesity and the correspondingly lower physical activity in Catania was expected on the basis of the available epidemiological studies [17]. There was a higher number of smoking parents in Catania than in Milano but the difference was lower among mothers. The median pre-pregnancy weight (59 *vs.* 58 kg) and the median weight increase during pregnancy (12 *vs.* 12 kg) were similar in Milano and Catania.

### 3.3. Effect of Place on the Anthropometry of NI636 Children

There was no statistically significant between-place difference in any anthropometric dimension except for the P_75_ of length (quantile regression models not shown). However, this difference (−1.0 cm for Milano *vs.* Catania, *p* < 0.05) is clearly negligible on biological grounds as it amounts to 1% of the corresponding percentile.

### 3.4. Effect of Place on the Energy and Nutrient Intake of NI636 Children

The between-place differences in the P_25_, P_50_ and P_75_ of energy and nutrient intake are given in Table 2.

**Table 2:** Difference in daily energy and nutrient intake between Milano and Catania.

Nutrient	ΔP_50_	ΔP_25_	ΔP_75_
E (kcal)	−17 (−2%)	−20 (−3%)	−2 (0%)
E (kcal·kg·weight^−1^)	−3 (−4%)	2 (3%)	−7 (−8%)
CHO (% E)	−4 * (−7%)	−3 * (−6%)	−3 * (−5%)
CHOS (% CHO)	−3 * (−8%)	−1 (−3%)	−1 (−2%)
FAT (% E)	2 * (6%)	3 * (10%)	2 * (6%)
SFA (% FAT)	1 (3%)	1 (3%)	−1 (−3%)
PRO (% E)	1 * (7%)	1 * (8%)	1 * (6%)
PRO (g·kg·weight^−1^)	0.2 (7%)	0.2 (10%)	0.1 (3%)
Sodium (mg)	−17 (−2%)	17 (6%)	6 (1%)
Iron (mg)	−1.4 * (−27%)	−0.5 * (−15%)	−2.0 * (−29%)
Fiber (g)	1.1 * (16%)	0.8 * (15%)	1.6 * (18%)

* *p* < 0.05 for Milano *vs.* Catania. Values are point estimates from multivariable quantile regression using age, sex and place as predictors (see *Statistical analysis*). The value between parentheses is obtained by dividing the point estimate by the median value of the corresponding intake in the pooled sample and is considered biologically relevant when greater than 10% (see *Sample Size*). Abbreviations: CHO = carbohydrates; CHOS = simple carbohydrates; ΔP_25_= difference in the 25th percentile between Milano and Catania; ΔP_50_ = difference in the 50th percentile between Milano and Catania; ΔP_75_ = difference in the 75th percentile between Milano and Catania; E = energy; FAT = fats; PRO = proteins; SFA = saturated fatty acids.

The median daily energy intake, *i.e.*, the main outcome, was −17 (95% CI −67 to 32) kcal lower in Milano than in Catania, well below the value that we considered biologically relevant, *i.e.*, 100 kcal (see *Sample Size*). This finding is coherent with the similar anthropometric status (see *Effect of Place on the Anthropometry of NI636 Children*), as anthropometry is a long-term indicator of energy balance [27]. According to our pre-specified threshold of 10% (see *Sample Size*), only the differences in iron intake (−27% at P_50_ in Milano) and fiber intake (+16% at P_50_ in Milano) could be considered biologically relevant. Interestingly, the differences detected at P_25_ and P_75_ were consistent with those detected at P_50_.

### 3.5. Effect of Place on the Energy and Nutrient Intake of NI636 Children

Due to the fact that it was only the amount of fiber and iron that differed in a biologically relevant way between Milano and Catania and there was no effect of place on anthropometry (see *Effect of Place on the Anthropometry of NI636 Children*), we removed place from the predictors of the quantile regression models and developed percentiles only on the basis of age and sex (quantile regression models not shown). Table 3, Table 4, Table 5, Table 6, Table 7 and Table 8 report the percentiles of anthropometry and nutrient intake estimated by applying such models.

Table 8 contrasts the nutrient intake of all the NI636 children with the Italian reference values [32].

**Table 3:** Percentiles of weight and length in the pooled sample of NI636 children.

Anthropometry		Weight (kg) (*n* = 390)			Weight (SDS) (*n* = 390)			Length (cm) (*n* = 268)			Length (SDS) (*n* = 268)		
		P_50_ *	P_25_ *	P_75_	P_50_	P_25_	P_75_	P_50_ *	P_25_ *	P_75_ *	P_50_	P_25_	P_75_
F	6 mo	7.6	7.0	8.3	0.38	−0.35	1.05	67.5	64.9	68.9	0.34	−0.70	1.37
M	6 mo	8.0	7.5	8.6	0.18	−0.48	0.78	68.5	67.0	70.0	0.16	−0.53	1.05
F	9 mo	8.6	7.9	9.4	0.37	−0.33	1.04	71.0	68.5	73.2	0.31	−0.69	1.25
M	9 mo	9.0	8.4	9.7	0.17	−0.47	0.78	72.0	70.6	74.3	0.13	−0.52	0.93
F	12 mo	9.4	8.7	10.3	0.35	−0.32	1.04	74.5	72.1	76.9	0.28	−0.68	1.13
M	12 mo	9.8	9.2	10.6	0.15	−0.45	0.78	75.5	74.2	78.0	0.10	−0.51	0.81
F	18 mo	10.9	10.0	11.9	0.33	−0.29	1.04	81.5	79.2	83.0	0.22	−0.67	0.89
M	18 mo	11.3	10.5	12.1	0.13	−0.43	0.78	82.5	81.3	84.1	0.04	−0.50	0.57
F	24 mo	12.0	11.1	13.1	0.30	−0.27	1.04	-	-	-	-	-	-
M	24 mo	12.4	11.6	13.4	0.10	−0.40	0.77	-	-	-	-	-	-
F	36 mo	14.0	12.9	15.3	0.25	−0.22	1.03	-	-	-	-	-	-
M	36 mo	14.4	13.4	15.6	0.05	−0.35	0.77	-	-	-	-	-	-

* *p* < 0.05 for sex. Values are point estimates from multivariable quantile regression using age and sex as predictors (see *Statistical Analysis*). Abbreviations: F = females; M = males; mo = month; P_25_ = 25th percentile; P_50_ = 50th percentile; P_75_ = 75th percentile; SDS = standard deviation scores.

**Table 4:** Percentiles of height and body mass index in the pooled sample of NI636 children.

Anthropometry		Height (cm) (*n* = 122)			Height (SDS) (*n* = 122)			BMI (kg/m^2^) (*n* = 390)			BMI (SDS) (*n* = 390)		
		P_50_	P_25_	P_75_	P_50_	P_25_	P_75_	P_50_	P_25_	P_75_	P_50_	P_25_	P_75_
F	6 mo	-	-	-	-	-	-	17.1	16.0	18.1	0.23	−0.82	0.89
M	6 mo	-	-	-	-	-	-	17.3	16.1	18.2	0.11	−1.06	0.76
F	9 mo	-	-	-	-	-	-	17.0	15.9	17.9	0.23	−0.48	0.88
M	9 mo	-	-	-	-	-	-	17.1	16.0	18.0	0.11	−0.71	0.76
F	12 mo	-	-	-	-	-	-	16.8	15.8	17.7	0.23	−0.36	0.87
M	12 mo	-	-	-	-	-	-	16.9	15.9	17.8	0.11	−0.60	0.75
F	18 mo	-	-	-	-	-	-	16.5	15.6	17.3	0.23	−0.27	0.86
M	18 mo	-	-	-	-	-	-	16.6	15.6	17.4	0.11	−0.51	0.74
F	24 mo	87.2	84.9	90.0	0.41	−0.40	1.19	16.1	15.3	17.0	0.23	−0.25	0.85
M	24 mo	88.0	86.4	91.0	0.22	−0.27	1.15	16.3	15.4	17.1	0.11	−0.48	0.73
F	36 mo	95.0	91.8	98.0	0.05	−0.81	0.82	15.5	14.9	16.2	0.23	−0.22	0.83
M	36 mo	95.8	93.3	99.0	−0.14	−0.68	0.78	15.6	14.9	16.3	0.11	−0.46	0.71

*p* > 0.05 for sex at all percentiles. Values are point estimates from multivariable quantile regression using age and sex as predictors (see *Statistical Analysis*). Abbreviations: BMI = body mass index; F = females; M = males; mo = month; P_25_ = 25th percentile; P_50_ = 50th percentile; P_75_ = 75th percentile; SDS = standard deviation scores.

**Table 5:** Percentiles of energy and carbohydrate intake in the pooled sample of NI636 children.

Nutrients		E (Kcal) (*n* = 390)			E (Kcal·kg·weight^−1^) (*n* = 390)			CHO (% E) (*n* = 390)			CHOS (% CHO) (*n* = 390)		
		P_50_ *	P_25_ *	P_75_ *	P_50_	P_25_	P_75_	P_50_	P_25_	P_75_	P_50_	P_25_	P_75_
F	6 mo	354	213	472	48	30	75	57	52	61	39	25	48
M	6 mo	419	289	545	51	37	74	57	52	62	42	27	49
F	9 mo	600	460	776	69	54	88	56	52	60	39	31	48
M	9 mo	665	536	849	72	60	87	56	52	61	42	33	48
F	12 mo	747	608	928	76	62	93	56	52	60	39	33	48
M	12 mo	812	684	1001	79	69	92	56	52	60	42	35	48
F	18 mo	921	783	1080	81	68	96	55	51	58	39	34	47
M	18 mo	986	859	1153	84	74	95	55	51	58	42	36	47
F	24 mo	1025	887	1156	83	70	97	54	50	56	39	35	46
M	24 mo	1090	963	1229	86	77	96	54	50	57	42	37	46
F	36 mo	1148	1011	1232	84	71	98	52	48	54	39	35	44
M	36 mo	1213	1087	1305	87	78	97	52	48	54	42	37	44

* *p* < 0.05 for sex. Values are point estimates from multivariable quantile regression using age and sex as predictors (see *Statistical analysis*). Abbreviations: CHO = carbohydrates; CHOS = simple carbohydrates; E = energy; F = females; M = males; mo = month; P_25_ = 25th percentile; P_50_ = 50th percentile; P_75_ = 75th percentile.

**Table 6:** Percentiles of fat and protein intake in the pooled sample of NI636 children.

Nutrients		FAT (% E) (*n* = 390)			SFA (% FAT) (*n* = 390)			PRO (% E) (*n* = 390)			PRO (g·kg·Weight^−1^) (*n* = 390)		
		P_50_	P_25_	P_75_	P_50_	P_25_	P_75_	P_50_	P_25_	P_75_ *	P_50_	P_25_	P_75_
F	6 mo	30	27	36	30	26	34	11	9	12	1.2	0.7	1.9
M	6 mo	30	27	36	31	26	35	11	10	13	1.4	0.9	2.1
F	9 mo	30	27	35	34	30	37	13	11	14	2.3	1.8	2.9
M	9 mo	30	27	35	35	30	39	13	12	15	2.5	2.0	3.1
F	12 mo	30	27	34	35	33	39	14	12	15	2.7	2.2	3.3
M	12 mo	30	27	34	37	32	40	14	12	16	2.9	2.4	3.4
F	18 mo	31	28	34	37	35	41	15	13	16	3.0	2.5	3.5
M	18 mo	31	28	34	39	34	42	15	14	17	3.2	2.7	3.7
F	24 mo	32	29	34	38	36	42	16	14	16	3.1	2.6	3.6
M	24 mo	32	29	34	40	36	43	16	14	18	3.3	2.8	3.7
F	36 mo	33	30	34	39	37	42	16	14	17	3.1	2.7	3.7
M	36 mo	33	30	34	40	37	44	16	14	18	3.3	2.9	3.8

* *p* < 0.05 for sex. Values are point estimates from multivariable quantile regression using age and sex as predictors (see *Statistical analysis*). Abbreviations: E = energy; F = females; FAT = fats; M = males; mo = month; P_25_ = 25th percentile; P_50_ = 50th percentile; P_75_ = 75th percentile; PRO = proteins; SFA = saturated fatty acids.

**Table 7:** Percentiles of iron, sodium and fiber intake in the pooled sample of NI636 children.

Nutrients		Iron (mg) (*n* = 390)			Sodium (mg) (*n* = 390)			Fiber (g) (*n* = 390)		
		P_50_	P_25_ *	P_75_	P_50_	P_25_ *	P_75_	P_50_	P_25_	P_75_
F	6 mo	1.8	1.2	4.3	75	16	157	3.5	1.9	5.6
M	6 mo	2.1	1.6	4.5	145	79	233	4.0	2.3	6.2
F	9 mo	4.0	2.6	6.1	360	247	493	6.0	4.5	7.4
M	9 mo	4.3	3	6.3	431	310	569	6.5	4.9	8.0
F	12 mo	4.8	3.3	6.7	563	411	732	6.9	5.4	8.3
M	12 mo	5.1	3.8	6.9	633	473	808	7.4	5.8	8.9
F	18 mo	5.4	4.0	7.2	848	641	1068	7.5	6.1	9.2
M	18 mo	5.7	4.5	7.4	918	704	1144	8.1	6.5	9.8
F	24 mo	5.6	4.4	7.4	1050	805	1307	7.8	6.3	9.6
M	24 mo	5.8	4.8	7.6	1121	868	1383	8.3	6.7	10.2
F	36 mo	5.7	4.8	7.5	1336	1036	1643	7.9	6.5	10.1
M	36 mo	6.0	5.2	7.7	1406	1098	1719	8.5	6.9	10.7

* *p* < 0.05 for sex. Values are point estimates from multivariable quantile regression using age and sex as predictors (see *Statistical analysis*). Abbreviations: F = females; M = males; mo = month; P_25_ = 25th percentile; P_50_ = 50th percentile; P_75_ = 75th percentile.

**Table 8:** Nutrient intake of the NI636 children compared with Italian reference values (*n* = 390).

Reference value	Frequency within Age Class (%)					
	6 mo	9 mo	12 mo	18 mo	24 mo	36 mo
CHO < 45% E (RI-LL)	3	5	4	2	5	10
CHO > 60% E (RI-UL)	32	24	17	4	11	0
CHOS < 15% E (SDT)	32	13	7	9	3	9
FAT < 40% E (AI)	87	94	-	-	-	-
FAT < 35% (RI-LL)	-	-	79	91	78	60
FAT < 40% (RI-UL)	-	-	93	100	97	90
SFA < 10% E (SDT)	49	53	33	16	22	9
PRO ≥ 1.32 g·kg·weight^−1^ (PRI)	54	84	-	-	-	-
PRO ≥ 1.00 g·kg·weight^−1^ (PRI)	-	-	100	100	100	100
Iron ≥ 11 mg (PRI)	0	0	-	-	-	-
Iron ≥ 8 mg (PRI)	-	-	28	21	19	14
Sodium > 1000 mg (UL)	-	-	-	43	66	79
Fiber > 8.4 g·kcal·E^−1^	-	-	52	38	42	33

Abbreviations: AI = average intake; CHO = carbohydrates; CHOS = simple carbohydrates; E = energy; FAT = fats; LL = lower limit; mo = month; PRI = population reference intake; PRO = proteins; RI = reference intake; SDT = suggested dietary target; SFA = saturated fatty acids; UL = upper limit.

### 3.6. Effect of between-Place Breastfeeding Frequency and between-Place Age Distribution on the Nutrient Intake and Anthropometry of NI636 Children

We fitted two additional multivariable quantile regression models to control for the potentially confounding effect of between-place breastfeeding frequency and between-place age distribution*.* Firstly, although the age-adjusted odds of breastfeeding was similar in Milano and Catania (see *Characteristics of NI636 Children*), we nonetheless tested whether there was an association between being breastfed in a given place and the outcomes of interest. To this aim, we added two covariates to the multivariable quantile regression models described under *Statistical Analysis* and reported above. Such covariates were breastfeeding (discrete: 0 = no; 1 = yes) and a breastfeedingXplace interaction (discreteXdiscrete). The main effect place (discrete: 0 = Catania; 1 = Milano) was already inside the models. The breastfeedingXplace interaction was not significant in all cases (*p* > 0.05). Secondly, although age had a similar distribution in Milano and Catania (see *Characteristics of NI636 Children*), we nonetheless tested whether there was an association between having a given age in a given place and the outcomes of interest. To this aim, we added a covariate to the multivariable quantile regression models described under *Statistical Analysis* and reported above. Such covariate was an ageXplace (continuousXdiscrete) interaction. The main effects age (continuous: 6, 9, 12, 18, 24 and 36 months) and place (discrete: 0 = Catania; 1 = Milano) were already inside the models. Age inside the ageXplace interaction was modeled using the same FP selected for age outside the interaction. The ageXplace interaction was not significant in all cases (*p* > 0.05).

## 4. Discussion

### 4.1. Main Findings

NI636 was aimed at comparing the intake of energy, macronutrients, fiber, sodium and iron and the anthropometric status of infants and toddlers living in Milano and Catania. Only the intake of iron and fiber differed in a biologically relevant way between Milano and Catania. Thus, contrarily to studies performed in convenience samples of Italian children ≥2 years [18,19], we did not detect an increasing north-to-south gradient of anthropometry and energy intake in the first 36 months of life. Despite the fact that anthropometry was within normal limits in most cases, NI636 children had a high intake of proteins, simple carbohydrates, saturated fatty acids and sodium, and a low intake of iron and fiber.

### 4.2. Strengths and Limitations

NI636 is the first study to report the macro- and micro-nutrient intake of children aged <12 months using the reference 7DWFR method and one of the very few studies that have employed a weighed food record in children from the general population [5]. A recent systematic review pointed out the need for using weighed food records to obtain more accurate estimates of nutrient intake in children [5]. Although a 3DWFR has recently been used to evaluate the changes of nutrient intake in infants taking part to a RCT [15,16], the 3DWFR is not as accurate as the 7DWR in quantifying nutrient intake and there is a great need of data to be obtained from the general pediatric population [6,7].

The main limitation of NI636 is that it was performed in a convenience sample. Judging from the higher school degrees and employment rates of NI636 parents compared to the general Italian population, it appears that we have selected an economically privileged sector of the population; therefore our findings are unlikely to generalize to the whole Italian population. We wish to point out, however, that we had no intention to select a representative sample of the general Italian population and we believe that our data represents an improvement over the current available data obtained mostly through surrogate methods in samples not drawn from the general population [5]. Another limitation of NI636 is that we did not estimate nutrient intake from breast milk. This is a choice that we did after having interviewed a random sample of mothers who uniformly expressed their concern for the need to regularly weigh their children before and after breastfeeding and because of the lack of reliable reference data for breast milk composition. However, we found no evidence that being breastfed in Milano *vs.* Catania had any effect on the outcomes of the study. This implies that the degree of underestimation of the nutrient intake of breastfed children will be comparable in Milano and Catania. A further limitation of NI636 is that no dietary assessment method can accurately estimate added salt in free living conditions. Thus, the sodium intake estimated by NI636 may be an underestimation of the true intake. Lastly, even if the 7DWFR is widely considered the dietary reference method [6,7], it is not without limitations [6,7,33]. For instance, more than 7 days of recording may be needed to accurately assess micronutrients [6,7]. However, the results from our data on micronutrient intake represent an improvement over existing data obtained at best with 3DWFR [5].

### 4.3. Anthropometry

Most of the NI636 children had normal values of weight for age, length/height for age and BMI for age (Table 3 and Table 4) [27]. For instance, the highest P_75_ of BMI was 0.89 SDS (6-month girls) (Table 4). Even if the energy and nutrient intake of breastfed NI636 children is underestimated because breast milk was not taken into account, the fact that median weight, length/height and BMI were within acceptable limits at all ages signals a good average nutritional status [27].

### 4.4. Proteins

The median intake of PRO increased from 1.2 to 3.3 g·kg·weight^−1^ from 6 to 36 months of age (Table 6). Even if the PRO intake of breastfed NI636 children is underestimated because the contribution of breast milk was not taken into account, there was a clear excess of PRO at all ages (Table 8). Observational studies have suggested that a high PRO intake may be a risk factor for later obesity and this hypothesis is currently being tested by RCTs [34]. We hope to be able to perform a follow-up study of NI636 children at about 5 to 7 years of age to test whether there is an association between PRO intake and more generally infant nutrition and later obesity [2,35].

### 4.5. Carbohydrates and Fiber

Most NI636 children had an intake of CHO within 45% and 60% of E, *i.e.*, within the limits suggested by the Italian guidelines [32] (Table 8). Although the fraction of E contributed by CHO decreased with age (Table 5), very few children at all ages had an intake of CHOS within the recommended value of 15% [32] (Table 8). Even if the present recommendations for CHO intake in children are extrapolated from adult data [32], the STRIP RCT has shown that reducing CHOS helps lower triglycerides in children [36]. STRIP has also shown the added benefits of a fiber-rich diet on triglycerides [36]. Using the threshold of 8.4 g·1000 kcal·E^−1^ suggested by the Italian guidelines, from 33% to 52% of NI636 children aged ≥12 months had an insufficient intake of fiber [32].

### 4.6. Fats

Despite the fact that the fraction of E contributed by FAT was nearly stable from 6 to 36 months (Table 6), there was a clear age-associated increase in SFA as percentage of FAT (Table 6) and most of the children were well above the limit of 10% of E suggested by the Italian guidelines (Table 8) [32].

### 4.7. Sodium

Although it is agreed that sodium intake should be limited during infancy because of its possible association with later cardiovascular disease [13,14], from 43% to 79% of NI636 children aged ≥12 months were consuming more sodium than the daily upper limit of 1000 mg defined by the Italian guidelines (Table 8) [32]. This is even more important in view of the fact that added salt may have escaped accurate quantification by the 7DWFR.

### 4.8. Iron

Iron-deficiency is the most common cause of anemia in infants and toddlers [12] and an evaluation of iron intake performed with reference methods is especially important in the first three years of life [5]. Using the population reference intake (PRI) as cut-point, as suggested by the Italian guidelines [32], very few children had a satisfactory iron intake. PRI has the obvious limitation that a child with a physiological requirement under the 97.5th percentile of the reference distribution will be classified as nutrient-deficient. However, PRI is the metric presently suggested for population studies and, according to this metric, most of our children had un unsatisfactory intake of iron [32].

## 5. Conclusions

NI636 is the first study to report the macro- and micro-nutrient intake of children aged <12 months using the reference 7-day weighed food record and one of the very few studies that have employed such reference method in children from the general population. NI636 showed that infants and toddlers living in Milano and Catania have a similar anthropometric status and nutrient intake with the exception of iron and fiber intake. They have a high intake of proteins, simple carbohydrates, saturated fats and sodium and a low intake of iron and fiber.

## Appendix

**Table A1:** Within-age between-pediatrician distribution of NI636 children.

Pediatrician	6 mo	9 mo	12 mo	18 mo	24 mo	36 mo
CT1	16%	8%	9%	12%	5%	7%
CT2	11%	10%	11%	4%	5%	5%
CT3	3%	11%	8%	3%	3%	3%
CT4	5%	16%	12%	13%	19%	9%
CT5	2%	3%	4%	3%	3%	5%
CT6	3%	8%	8%	12%	9%	12%
CT7	2%	3%	7%	7%	6%	2%
MI1	0%	0%	0%	0%	5%	16%
MI2	19%	21%	24%	27%	33%	16%
MI3	32%	16%	13%	16%	11%	24%
MI4	8%	3%	4%	3%	2%	2%
Total	100%	100%	100%	100%	100%	100%

Values are percentages within age classes. Percentages may not total 100 because of rounding. Abbreviations: MI = Milano; CT = Catania.
